# Key Technologies in Developing Chip-Scale Hot Atomic Devices for Precision Quantum Metrology

**DOI:** 10.3390/mi15091095

**Published:** 2024-08-29

**Authors:** Huiyao Yu, Xuyuan Zhang, Jian Zhang, Zhendong Wu, Long Jiao, Kan Li, Wenqiang Zheng

**Affiliations:** 1 Zhejiang Provincial Key Laboratory and Collaborative Innovation Center for Quantum Precision Measurement, College of Science, Zhejiang University of Technology, Hangzhou 310023, China; yuhuiyao@zjut.edu.cn (H.Y.); 2112109017@zjut.edu.cn (X.Z.); 17502348285@163.com (J.Z.); 211123090029@zjut.edu.cn (Z.W.); 2 College of Mechanical Engineering, Zhejiang University of Technology, Hangzhou 310023, China; jiaolong@zjut.edu.cn

**Keywords:** chip-scale, hot atomic devices, MEMS cells, VCSEL lasers, atomic magnetometers, atomic gyroscopes, atomic clocks

## Abstract

Chip-scale devices harnessing the interaction between hot atomic ensembles and light are pushing the boundaries of precision measurement techniques into unprecedented territory. These advancements enable the realization of super-sensitive, miniaturized sensing instruments for measuring various physical parameters. The evolution of this field is propelled by a suite of sophisticated components, including miniaturized single-mode lasers, microfabricated alkali atom vapor cells, compact coil systems, scaled-down heating systems, and the application of cutting-edge micro-electro-mechanical system (MEMS) technologies. This review delves into the essential technologies needed to develop chip-scale hot atomic devices for quantum metrology, providing a comparative analysis of each technology’s features. Concluding with a forward-looking perspective, this review discusses the future potential of chip-scale hot atomic devices and the critical technologies that will drive their advancement.

## 1. Introduction

Laser-based atomic techniques have proven to be valuable tools for precision measurements in various scientific and technological applications. Alkali metal atoms, in particular, are often employed in these techniques due to their unique properties that make them effective measurement media. In accordance with distinct temperature states, atomic ensembles utilized in a specific experiment or system can be broadly categorized into cold atoms and hot atoms. Each temperature regime has its own advantages and applications. Researchers make the choice between hot and cold atomic ensembles based on the requirements of their experiments and the specific properties they wish to exploit. Broadly speaking, quantum technology relying on hot atomic ensembles is more advanced in terms of practical applications, signifying a more mature stage of development. This is attributed to the ease of handling and manipulation associated with hot atomic systems. Specialized hot atomic devices, including atomic magnetometer [1,2,3,4], atomic clock [5,6,7,8], atomic gyroscope [9,10,11,12], and Rydberg-atom-based electrometry [13,14,15,16], have been successfully developed and are now transitioning into the practical application phase.

Atomic devices possess numerous advantages that traditional precision measurement technologies cannot rival, and these advantages may stem from multiple aspects. In the case of magnetometers, for example, superconducting quantum interference devices (SQUIDs), which have been the most sensitive magnetic sensors of the last decades, obtain the magnitude of the magnetic field by measuring the magnetic flux of a loop formed by two Josephson junctions. Low-T_C_ SQUIDs can achieve a sensitivity as high as 1 fT/Hz^1/2^ [17]. However, SQUIDs require ultra-low temperatures to maintain the superconducting state, making their probe systems typically bulky and dependent on a constant supply of coolant. This requirement significantly increases the cost of these magnetic field measurement devices. Fluxgate magnetometers are commonly used in field operations due to their portability, but the sensitivity of a typical fluxgate is limited to around 100 pT/Hz^1/2^ [18]. Inductive coil sensors, known for their high sensitivity at high frequencies, are widely used in high-field magnetic resonance. However, their sensitivity increases linearly with frequency, making them less effective in the low-frequency region [19]. In recent years, advancements in atomic magnetometer technology have positioned them as the most sensitive magnetic field detection devices, surpassing SQUIDs. Unlike SQUIDs, atomic magnetometers have the advantage of not requiring cryogenic operation. Over the past decades, significant efforts have been made to enhance the sensitivity of atomic magnetometers. As a result, the sensitivity of SERF magnetometers and RF magnetometers can now achieve levels below 1 fT/Hz^1/2^ [1].

The quantum metrology research community, centered around hot atomic ensembles, is at the forefront, actively working towards the development of chip-scale hot atomic devices. Here, we review the domain of chip-scale hot atomic devices within the framework of quantum metrology. A significant portion of the review is devoted to introducing key technologies driving the realization of chip-scale hot atomic devices, shedding light on their existing status and prospects.

Atomic sensors, while diverse in application and control schemes, rely on several core technologies that define their performance. Central to these sensors are gaseous alkali atoms, contained within high-quality vapor cells. The performance of these cells often sets the performance ceiling for the entire device. As light–atom interactions are essential for the atomic sensor’s operation, optical systems also play a crucial role. The rapid advancement of atom-based quantum precision measurement technology owes much to breakthroughs in laser technology, although the existing capabilities of lasers also limit these developments to some extent. Selecting a proper light source is critical for miniaturizing atomic devices. Beyond optical interactions, atomic sensors also utilize magnetic fields to manipulate atomic spins, typically generated by coils that provide both static and alternating magnetic fields. Furthermore, temperature control, achieved through electrically heated coils, regulates the number of atoms participating in measurements. Miniaturizing these subsystems without compromising their performance is crucial for the development of chip-scale atomic devices.

In this paper, we review the key technologies essential for chip-scale hot atomic devices within the realm of quantum metrology from the perspective of each subsystem. Our focus encompasses the fabrication of vapor cells, the development of laser sources, and the design of coil systems.

## 2. Vapor Cells

The atomic vapor cell, as the core component of hot atomic devices, plays a crucial role in ultimately determining the overall system performance. In today’s trend towards miniaturization and portability, reducing vapor cell size while maintaining performance has become a significant research topic in the field of hot atomic devices. This challenge demands continuous optimization and innovation in existing manufacturing processes, as well as new breakthroughs in materials science and microfabrication [20,21,22,23].

### 2.1. Glass-Blowing Technique

Before introducing the preparation of miniaturized atomic vapor cells, let us review the technique of vapor cell fabrication using glass blowing [24]; all preparation methods for miniaturized atomic vapor cells have, to some extent, drawn upon the experience of this fabrication technology.

**Figure 1:**
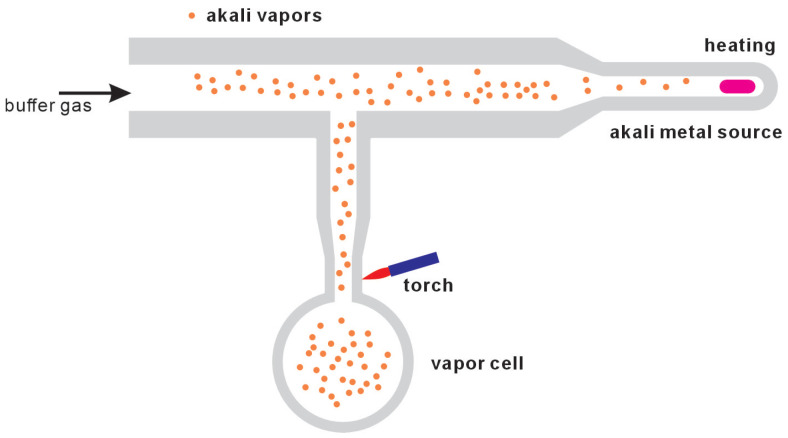
Glass vapor cells and fabrication equipment.

In the initial stage of the preparation process, all components are rigorously evacuated to a high vacuum of approximately $10^{-5}$ Torr. However, many researchers aim for even higher vacuum levels, up to $10^{-9}$ Torr, to further minimize the influence of impurities within the vacuum system. Subsequently, as shown in Figure 1, either a single buffer gas or a carefully calibrated mixture of buffer gases is introduced into the glass tube and the internal cell. Alkali metal vapor is produced by either evaporating pure liquid metal or, more commonly, dispensing alkali atoms from a wire dispenser—the latter method being favored for its convenience and efficiency. These vapors then diffuse into the cell, which is maintained at ambient temperature, and condense inside, forming tiny droplets. Upon completion of these steps, a specialized gas torch is used to precisely sever the connecting glass capillary, effectively isolating the cell from the rest of the glass structure in a clean break.

The glass-blowing technology for atomic vapor cells is widely utilized and has yielded excellent results. However, its direct application to chip-scale hot atomic devices poses challenges. The issue is not solely the difficulty in miniaturizing the vapor cells, as the technology for crafting cubic glass vapor cells measuring 2 mm × 2 mm × 2 mm is already relatively mature. A more significant concern is that in mass production, this method exhibits limitations in consistency and productivity, and it struggles with compatibility within on-chip integration processes.

### 2.2. MEMS Vapor Cell Fabrication

**Figure 2:**
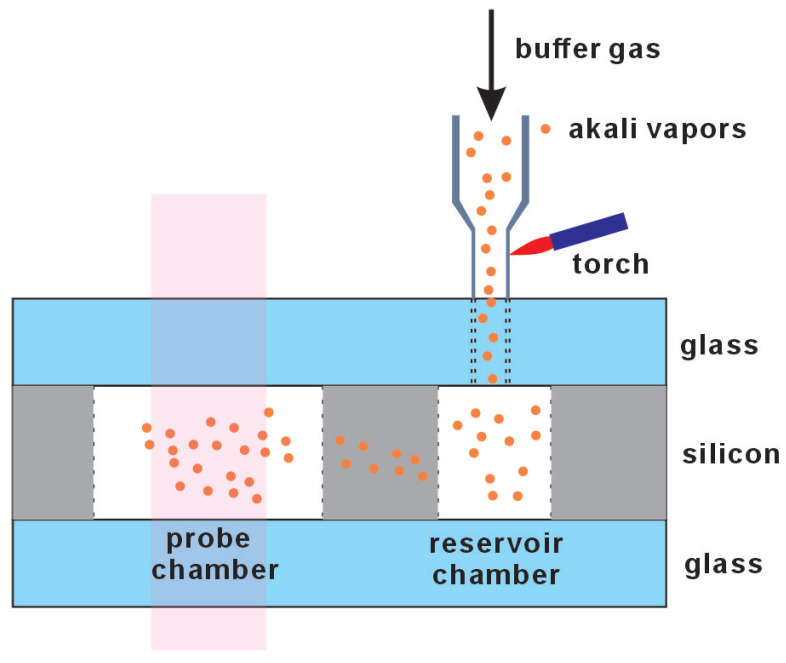
Combination of glass blowing and microfabrication. Liquid alkali metal is introducted into microfabricated vapor cell with a small glass tube and cut off with a gas torch.

MEMS vapor cells are the most prevalent form of miniaturized atomic vapor cells. These cells typically feature a glass–silicon–glass packaging structure, as shown in Figure 2. Alkali atoms are housed within a cavity etched into a silicon wafer, with glass wafers bonded to both the top and bottom surfaces to create a hermetic seal. The glass windows permit light to enter the cavity and interact with the atoms. Utilizing MEMS technology to prepare atomic vapor cells presents several benefits. First, it ensures good consistency. A batch of vapor cells can be simultaneously produced on the same silicon wafer, maintaining uniform process conditions. Second, etching technology allows for the creation of atomic cavities with sub-micron dimensions and the ability to construct complex, customized cavity structures. Lastly, the resulting vapor cells have a regular geometric structure without the side tubes common in traditional glass-blown cells, facilitating straightforward integration with optical, optoelectronic, and electronic components.

#### 2.2.1. Introduction of Alkali Atoms and Gases

Like the glass-blowing technique used to fabricate vapor cells, the preparation of a MEMS vapor cell involves confining reactive alkali metal atoms within a sealed cavity to shield them from contaminants such as H_2_O and O_2_. This process may also require the addition of a buffer gas, such as He or N_2_, if necessary. Therefore, the methods used to inject highly reactive alkali metals into the cell and maintain a stable inert gas environment are crucial steps in the preparation process.

##### Pure Alkali Metal

The introduction method commonly employed in glass-blowing technology can also be adapted in the MEMS vapor cell, as depicted in Figure 2 [25]. Initially, the cell cavity is constructed using MEMS technology, designed internally with two distinct compartments: a reservoir cell and a probe cell. An aperture is created in the reservoir cell, to which a thin tube, connected to an external vacuum line used in glass-blowing technology, is attached. Following this setup, alkali metal is allowed to diffuse into the cell, and the requisite buffer gas is introduced. The tube is then severed using a very small gas torch to finalize the seal. This approach not only ensures the purity of the alkali metal introduced but also allows for relatively precise control over the buffer gas pressure. However, the presence of the branch tube may compromise the flatness of the cell surface, potentially impacting the integration of additional devices later on. Additionally, scaling this method up for large-scale production could present some challenges.

**Figure 3:**
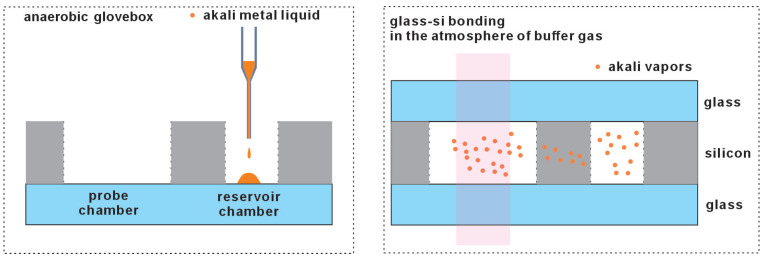
Liquid alkali metal is directly introduced via pipetting under an inert atmosphere.

Introducing liquid alkali metal via pipetting is an efficient method, as depicted in Figure 3 [26,27,28]. The alkali metal is stored in a sealed glass ampoule under an inert atmosphere. The ampoule must be broken within an anaerobic glovebox to maintain an oxygen-free environment, ensuring the metal’s purity. Once opened, the alkali metal can be drawn into a pipette. This facilitates its transfer to the glass–silicon preform. Before the final encapsulation, the required buffer gas can be introduced.

**Figure 4:**
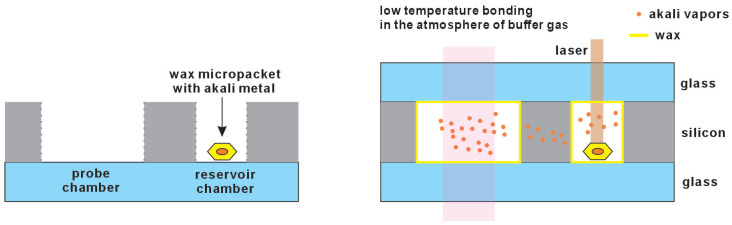
The alkali metal is introduced by a wax micropacket with alkali metal. After being sealed within a cell, the alkali metal can be released through laser heating.

Wax is a chemically inert material suitable for containing alkali metals. Utilizing micromachining techniques, a small quantity of alkali metal can be encapsulated in wax to create micropackets. These can then be sealed within a cell, with the alkali metal released through laser heating. The connection channel between the probe chamber and the reservoir chamber is fabricated using micromachining technology and is typically designed as multiple microchannels. This design prevents chemical reaction residues from entering the probe chamber, while allowing alkaline metal vapor to pass smoothly through the microchannels into the probe chamber. This method facilitates the formation of pure alkali metal vapor within the final vapor cells. This method yields precise amounts of alkali metal required for each type of vapor cell, ensuring consistent performance and minimizing the wastage of costly alkali metals. Additionally, the simplicity of this fabrication process and the ease of handling facilitate the cost-effective production of vapor cells. Once the vapor cell is sealed, the wax coating surrounding the alkali metal adheres to the inner wall, thereby reducing collisions between the alkali metal atoms and the cell’s interior surfaces, as shown in Figure 4 [29].

##### Chemical Reaction

Within the sealed vapor cell cavity, chemical reactions between an alkali metal chloride and barium azide are employed to produce alkali metal atoms. These materials are stable in the air, enhancing ease of handling. This approach can make the process flow simplified. The reactions to produce rubidium metal atoms can be described as follows [17,26,30]:

(1) $${\mathrm{BaN}}_{6}\overset{200\;{}^{\circ}C}{\to }\mathrm{Ba}+3N_{2}↑,$$

(2) $$2\mathrm{RbCl}+\mathrm{Ba}\overset{250–300\;{}^{\circ}C}{\to }2\mathrm{Rb}↑+\;{\mathrm{BaCl}}_{2}.$$

**Figure 5:**
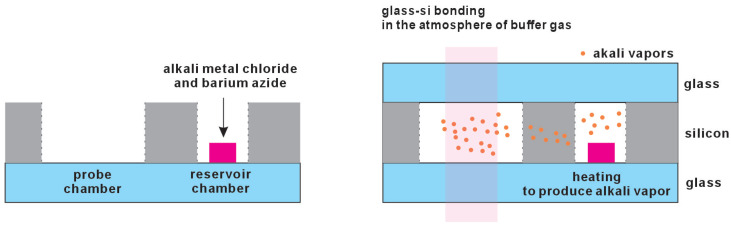
The alkali metal can be introduced by chemical reaction between an alkali metal chloride and barium azide. The alkali metal will be released through heating the reaction materials.

The process involves fabricating a glass–silicon preform, placing a mixture of alkali chloride and barium azide inside it, and finally encapsulating it in a buffer gas atmosphere, as depicted in Figure 5. Typically, during the encapsulation, preheating is applied to decompose the azide, which produces barium and nitrogen gas. If nitrogen is not the desired buffer gas, it is evacuated. Nitrogen gas produced from the reaction can serve as a buffer gas, potentially creating a large buffer gas pressure.

Additionally, alkali metal chloride and barium azide can be stored inside a glass ampoule equipped with a heater. Pure alkali metals are obtained by heating the ampoule. The alkali metal can then be transferred into the cell via pipetting. This method significantly simplifies the procedure compared to extracting pure alkali metals in an inert environment.

**Figure 6:**
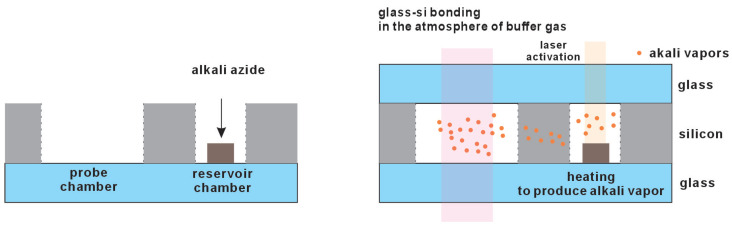
Another chemical reaction method uses alkali metal azide to introduce alkali metal. Ultraviolet light is used to catalyze and initiate the decomposition of the compound.

Another chemical reaction method involves using physical deposition to apply a layer of alkali metal azide or pipetting a solution of alkali metal azide onto the inner side of the cell window, as shown in Figure 6 [31,32]. This compound is relatively stable in air, making it easy to handle. After sealing the vapor cell, ultraviolet light (254 nm) is used to catalyze and initiate the decomposition of the compound, producing alkali metal and nitrogen gas inside the cell. Compared to the reaction between alkali metal chloride and barium azide under heated conditions, this process takes longer, typically several hours or more. Despite the extended reaction time, there may still be issues with the incomplete reaction of the reactants, potentially affecting the long-term stability of the vapor cell components.

##### Electrolytic Alkali Metal

**Figure 7:**
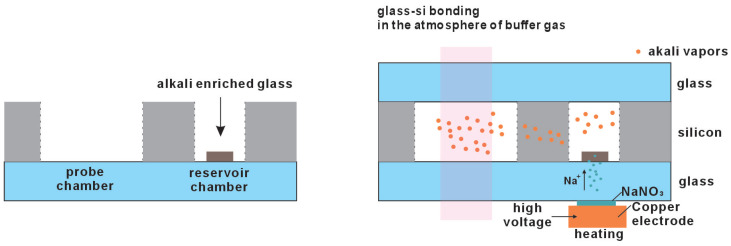
The alkali metal can be introduced by the method of electrolysis through reducing the alkali metal elements from the alkali-metal-enriched glass.

Electrolysis can also be employed to reduce alkali metal elements, as dipicted in Figure 7 [33]. Initially, glass materials containing desired alkali metal ions are prepared through diffusion. A small quantity of this alkali-metal-enriched glass is then encapsulated within the reservoir chamber of the vapor cell. The chemical stability of this glass ensures that no reactions occur during the high-temperature anodic bonding process, thus resulting in optimized bonding effects. After the vapor cell is sealed, molten sodium nitrate is applied to the window of the reservoir chamber for electrolysis. Under high temperature and voltage, sodium ions are driven towards the glass under the influence of the electric field, where they enter the alkali metal glass material, displacing the desired alkali metal ions and reducing them to the requisite alkali metal elements.

#### 2.2.2. The Bonding Techniques

Glass–silicon bonding is a crucial step in the fabrication of MEMS vapor cells, with anodic bonding being the most widespread technique [34,35]. Anodic bonding involves fusing silicon and glass wafers by applying high temperature and voltage. Under specific temperature and voltage conditions, sodium ions in the glass migrate towards the interface, creating a depletion layer, while electrons in the silicon wafer move in the opposite direction. This ion migration facilitates electrostatic attraction and results in a chemical bond at the contact interface between the silicon and glass wafers. The process is as follows: Initially, the surfaces of both silicon and glass wafers are meticulously cleaned and polished to enhance surface flatness, thereby improving bonding efficiency. Subsequently, the silicon and glass wafers are placed together and heated to above 300 °C, accelerating the migration of metal particles. A specific voltage, typically in the hundreds of volts range, is then applied to drive ion migration, achieving a stable bonded state. Finally, the bonded silicon–glass assembly is cooled to room temperature, completing the bonding process.

Conventional high-temperature anodic bonding technology is typically employed in the fabrication of atomic vapor cells containing buffer gas. However, certain applications require the application of anti-relaxation coatings to the inner walls of the cell to suppress wall relaxation and reduce hyperfine resonance linewidth. This is particularly important for the preparation of non-classical quantum states in hot atomic ensembles [36,37,38]. Common coating materials like paraffin and octadecyltrichlorosilane (OTS) have limited heat resistance and may degrade under the high temperatures used in bonding [39]. Consequently, various low-temperature bonding technologies have been developed to address this issue.

The low-temperature anodic bonding process utilizing lithium–niobium–phosphate glass layers is primarily employed for the encapsulation and connection of chip-level or micro-devices at lower temperatures. This method leverages the specific properties of lithium–niobium–phosphate glass, such as its high ion conductivity, to establish strong and hermetic bonds at temperatures significantly lower than those required by traditional high-temperature bonding methods. Operating at lower temperatures minimizes thermal stress and deformation, while still achieving robust bond strength and hermeticity, which are crucial for the reliability and stability of the encapsulation. Furthermore, the process is relatively simple and cost-effective, making it well-suited for large-scale production. By depositing lithium–niobium–phosphate glass layers on the bonding surfaces of the gas cells through RF sputtering, anodic bonding can be performed even at room temperature, effectively sealing the gas cells [40].

Indium bonding is a technique that employs the element indium to create a durable bond between two materials [41,42]. Known for its excellent electrical conductivity and thermal stability, indium bonding is frequently utilized in the microelectronics industry. The process involves placing a thin layer of indium on the bonding surfaces, followed by the application of heat and pressure. This causes the indium to flow and wet the surfaces, resulting in a robust bond. The low melting point of indium enables bonding at reduced temperatures, which minimizes the risk of thermal damage to sensitive materials. Utilizing indium bonding, it is feasible to package a vapor cell with an OTS coating at temperatures as low as 140 °C, effectively preserving the anti-relaxation properties of the coating.

Another bonding method involves low-temperature solder to bond glass to LTCC (low-temperature cofired ceramic) [43]. Eutectic solder alloys, which have a low melting point, enable the sealing process to be conducted at very low temperatures. This sealing occurs rapidly, typically within minutes, and features a peak temperature duration (solder melt time) of approximately 10 s. This brief exposure to heat reduces the evaporation of alkali metals and minimizes unwanted reactions. As a result, the joint is expected to remain stable and hermetically sealed.

Copper–copper thermal compression bonding is a technique that connects two copper surfaces using heat and pressure [44]. This method generally involves heating the copper surfaces to temperatures near the melting point of copper and then applying pressure to fuse them tightly. During the cooling process, strong metallic bonds form between the copper atoms, creating a durable and permanent connection. Previous reports have also mentioned the successful application of copper–copper thermal compression bonding technology in the manufacturing of gas cells.

#### 2.2.3. Cells for Dual-Beam Configurations

**Figure 8:**
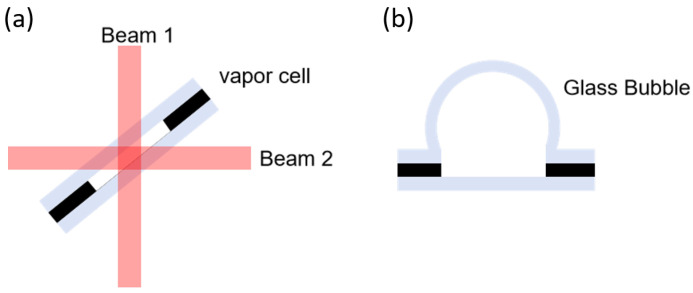
Microfabricated vapor cells for dual-beam configurations, (**a**) 45-degree incidence approach, (**b**) glass spherical vapor cell.

The vapor cell described earlier is packaged using anodic bonding, typically with silicon as the intermediate layer, resulting in only two transparent windows. However, some atomic devices require a dual-beam configuration, which can be achieved using a 45-degree incidence method [45], as illustrated in Figure 8a. Alternatively, in vapor cell manufacturing, glass can be used as the intermediate layer, with silicon wafers as the encapsulation layers on both sides, creating four transparent surfaces. Another approach is to use thermal expansion to shape the cover glass layer into a glass sphere [46], as shown in Figure 8b, providing multiple light-passing surfaces.

#### 2.2.4. Cell Insulation

The use of an insulation structure for the vapor cell can ensure stable temperature within the vapor cell and effectively reduce heating energy consumption. Typically, non-magnetic and high-temperature-resistant materials such as PEEK, silicon, and polyimide are used to construct a sealed chamber, which may even be a vacuum chamber. By placing the vapor cell inside this chamber, heat loss and fluctuations caused by air convection are reduced. Additionally, to minimize heat loss due to thermal conduction, it is important to minimize the contact area between the vapor cell and the chamber walls. This can be achieved, for example, by suspending the vapor cell within the chamber using a polyimide web, thereby reducing direct contact.

## 3. Lasers

The quality of the laser beams is critical in the system of light–atom interaction, playing a pivotal role in constructing an ultimate quantum precision measurement device. One of the fundamental reasons for the rise of atomic precision measurement technology is the rapid development of laser technology. Although the performance of lasers has greatly improved compared to decades ago, and their size has been continuously reduced, the chip-scale integration of lasers remains a significant challenge in the development of chip-scale atomic devices. The evaluation of laser quality should be multi-dimensional. Generally, atomic devices of precision measurement are strictly for beam quality, linewidth, frequency tunability, and frequency and power stability.

Different atomic precision measurement techniques prioritize different aspects of laser requirements. For example, atomic gyroscopes have high requirements for sufficient laser power, and optical atomic clocks require lasers with extremely high-frequency stability and ultra-narrow linewidths. In addition to performance parameters, the practicality in real-world applications is also an important basis for selecting lasers.

Semiconductor lasers, also known as laser diodes, are the most suitable laser sources for miniaturized hot atom devices among all laser types. These lasers utilize semiconductor materials as their active medium. They are compact, durable, and can be easily powered through simple current injection, making their operating voltage and current compatible with integrated circuits for potential monolithic integration. Numerous semiconductor manufacturing processes, such as secondary epitaxy, chemical vapor deposition, step photolithography, nanoimprinting, electronic etching, and ion etching, are directly applicable to the fabrication of semiconductor lasers. Laser diodes can be classified into two main types based on their configuration: edge-emitting laser diodes and surface-emitting laser diodes.

### 3.1. Edge-Emitting Laser Diodes

Edge-emitting laser diodes are the most technologically mature laser diodes with the most complete range of operating wavelengths. Their most important feature is that the direction of laser emission is perpendicular to the direction of semiconductor material growth. Originally, Fabry–Pérot (F–P) resonant cavities were used to provide optical feedback in laser diodes. In 1971, Bell Labs, USA, first proposed the idea of using Bragg grating structures instead of mirrors to provide feedback and analyzed the working principle of these feedback structures [47]. Then F–P resonant cavities were replaced by the distributed Bragg reflectors (DBRs). While providing optical feedback, DBRs also serve as mode-selective structures that allow the lasers to achieve full single-mode operation.

**Figure 9:**
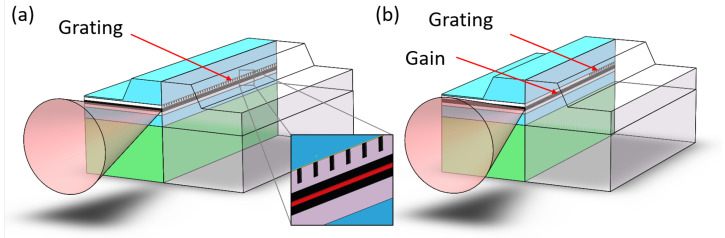
Schematic representation of the structures for (**a**) DFB and (**b**) DBR lasers.

The structures of two edge-emitting laser diodes that utilize Bragg grating structures are illustrated in Figure 9. The distributed feedback (DFB) laser integrates a Bragg grating with the active region and incorporates a phase-shift region in the middle to select the laser wavelength. DFB lasers resemble DBR lasers in their use of internal grating structures. However, the key difference lies in the configuration of the gratings: the grating in a DFB laser extends throughout the entire active (gain) region, while in a DBR laser, the grating is positioned outside the active region. Generally, DBRs offer the ability to integrate multiple regions, which is typically absent in DFBs, providing enhanced control and a broader tuning range. For instance, a multi-electrode DBR laser may include a phase control region that allows for independent tuning of the phase, separate from adjustments to the grating period and laser diode current. This capability enables DBRs to maintain single-frequency operation across an extensive tuning range. High-end sampling grating DBR lasers, for example, can be tuned over a 30–40 nm range. DBRs are also available in a simpler, single-electrode configuration, offering a reduced tuning range similar to that of DFB lasers, but being prone to current and temperature-induced mode hopping. Moreover, DBRs can handle a higher current input since the grating does not extend through the active area, typically resulting in slightly higher output power than DFBs, albeit with increased power, current noise, and consequent power jitter. DFB lasers provide a more integrated and unified structure. They effectively address the significant issues of wavelength drift and mode hopping seen in DBR structures, making them the most stable and practical laser cavity structure.

**Figure 10:**
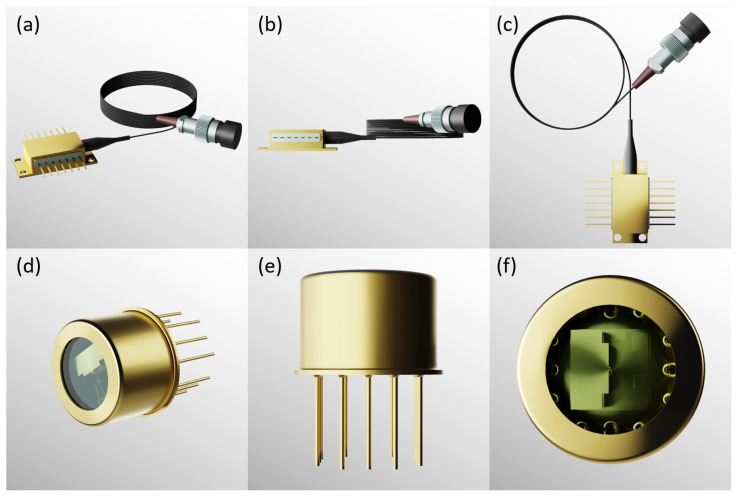
DBR laser diodes from Photodigm Co. (**a**) Perspective view, (**b**) right view, and (**c**) top view of a butterfly-package DBR laser diode. (**d**) Perspective view, (**e**) right view, and (**f**) top view of a TO-package DBR laser diode. Figure from [48].

Edge-emitting laser diodes used in hot atomic devices are now generally available as commercial products. Key manufacturers of DBR and DFB lasers, which are tuned to the Rb and Cs D_1_ and D_2_ lines, include Thorlabs (USA), Photodigm (USA), Toptica Eagleyard (Germany), and Sacher Lasertechnik (Germany), among others. The specifications for some commonly used commercial edge-emitting laser diodes are listed in Table 1. Figure 10 illustrates the morphology of the DBR laser diodes from Photodigm Co, which incorporate four internal components: a laser diode (LD), a photodiode (PD), a thermoelectric cooler (TEC), and a thermistor. This configuration enables the production of a diffraction-limited, single longitudinal-mode output laser beam.

### 3.2. Surface-Emitting Laser Diodes

**Figure 11:**
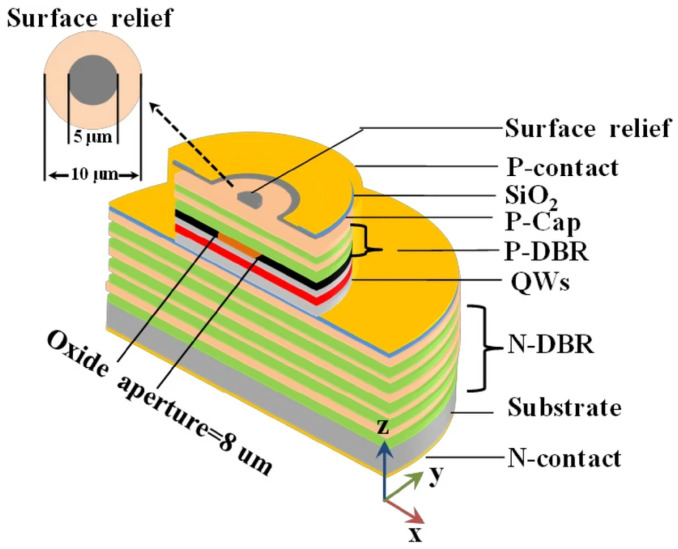
Schematic of VCSELs with oxide aperture and surface relief. Figure from [52].

In 1979, Soda et al. first proposed the concept of the vertical-cavity surface-emitting laser (VCSEL) [49], a type of semiconductor laser noted for its compactness, high efficiency, variety of operating wavelengths, ease of integration, reliability, and scalability [50,51]. The VCSEL’s structure, as shown in Figure 11, includes upper and lower DBRs, quantum wells (QWs), oxide apertures, and both upper and lower metal electrodes. Its active region, situated between the n-doped and p-doped DBRs, emits light perpendicular to the substrate.

VCSELs differ from traditional edge-emitting semiconductor lasers in several significant ways. Unlike edge-emitting lasers, which can only be tested post-fabrication, VCSELs can be assessed at any stage of production. This feature allows for the identification and resolution of issues like poor contact or material growth without wasting fabrication time and materials. In fact, tens of thousands of VCSELs can be processed simultaneously on a three-inch gallium arsenide chip due to their perpendicular emission relative to the reaction area, in contrast to the parallel emission of edge-emitting lasers. Although VCSEL production requires more precise labor and finer materials, the outcomes are more controllable and predictable [53,54]. Furthermore, VCSELs offer several key advantages over edge-emitting semiconductor lasers [55]:

1. Beam quality: VCSELs emit light from a circularly symmetric aperture, creating a spot with a small divergence angle. This symmetry facilitates simple, low-cost, and efficient coupling through basic beam shaping systems. In contrast, edge-emitting lasers produce elliptical spots with aspect ratios up to 100:1, often requiring additional beam shaping systems.
2. Low threshold: The small active region in VCSELs enables low threshold lasing, potentially down to the microampere level.
3. Absence of facet damage: The reflectors in VCSELs are epitaxially grown DBRs, which prevents optical damage to the laser cavity facets.
4. Stability and reliability: The refractive index of the semiconductor materials used in VCSELs varies minimally with temperature changes, leading to minimal wavelength drift. Additionally, VCSELs operate effectively across a broad temperature range.
5. Single-mode output: The resonant cavity length of VCSELs is very short, often just a few micrometers, with large separation between longitudinal modes. This typically results in a single longitudinal-mode output and a higher relaxation oscillation frequency.
6. Large-scale two-dimensional arrays: The perpendicular emission direction of VCSELs relative to the substrate facilitates the integration of large-scale two-dimensional arrays. This alignment also removes the need for cleaving, enabling low-cost fabrication and testing, as well as high-density integration with high power output.

**Figure 12:**
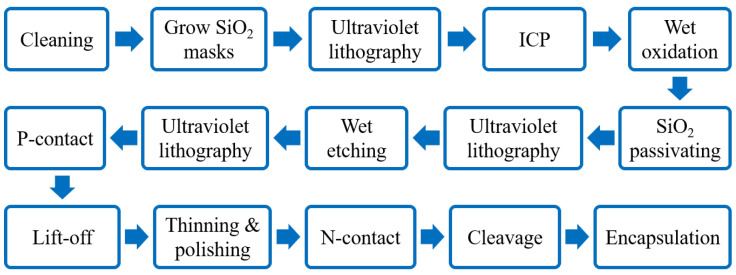
VCSEL preparation process. Figure from [57].

The fabrication process for VCSELs has developed into a comprehensive system. Take the large-aperture single-mode 795 nm VCSEL prepared by the Chinese Academy of Sciences as an example, a plasma-enhanced etching process is used to create a mesa with a diameter of 20 μm. Subsequently, a wet selective oxidation process is applied to form an oxide aperture with a diameter of 6 μm on an Al_0.98_Ga_0.02_As layer. A 300 nm SiO_2_ insulation layer is then deposited across the entire device surface using plasma-enhanced chemical vapor deposition. The electrical injection window is formed by etching the SiO_2_ layer located at the center of the mesa, with a window diameter of 16 μm. Finally, a metal layer is applied using magnetron sputtering, and a lift-off process is utilized to establish an emission aperture with a diameter of 10 μm [56]. Figure 12 also gives a generalized preparation method for VCSELs.

The advancement of chip-scale hot atomic devices has significantly increased the performance requirements for VCSELs, which are continually being innovated to meet these demands. As early as 2000, the University of Colorado and the National Institute of Standards and Technology (NIST) demonstrated the development of atomic clocks using VCSELs [58]. Later, NIST reported the use of a 795 nm VCSEL in a chip-scale atomic magnetometer [59]. Before entering the alkali metal vapor cell, the laser beam was conditioned through several optical elements to produce circular polarization and attenuate the light. The magnetometer’s vapor cell was filled with ^87^Rb and N_2_ at 270 kPa, resulting in an optical linewidth broadening to 18 GHz.

**Figure 13:**
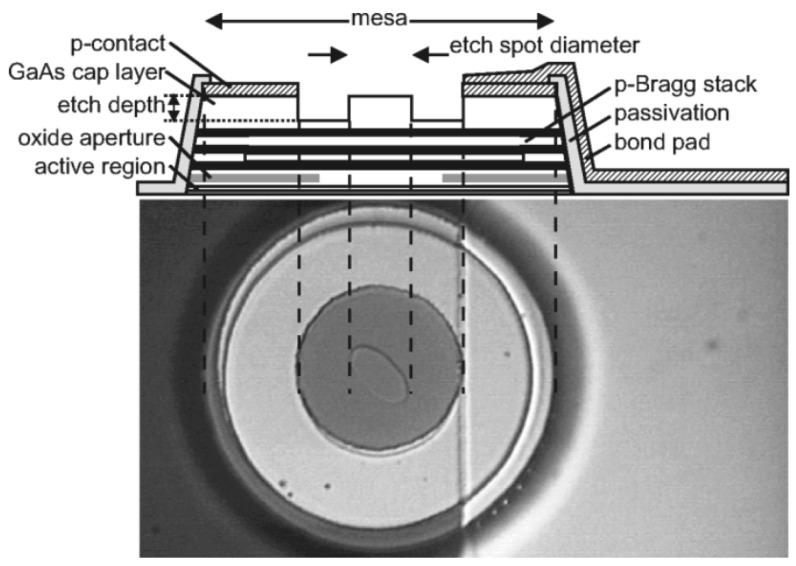
VCSEL fabricated by the University of Ulm. Figure from [60].

**Figure 14:**
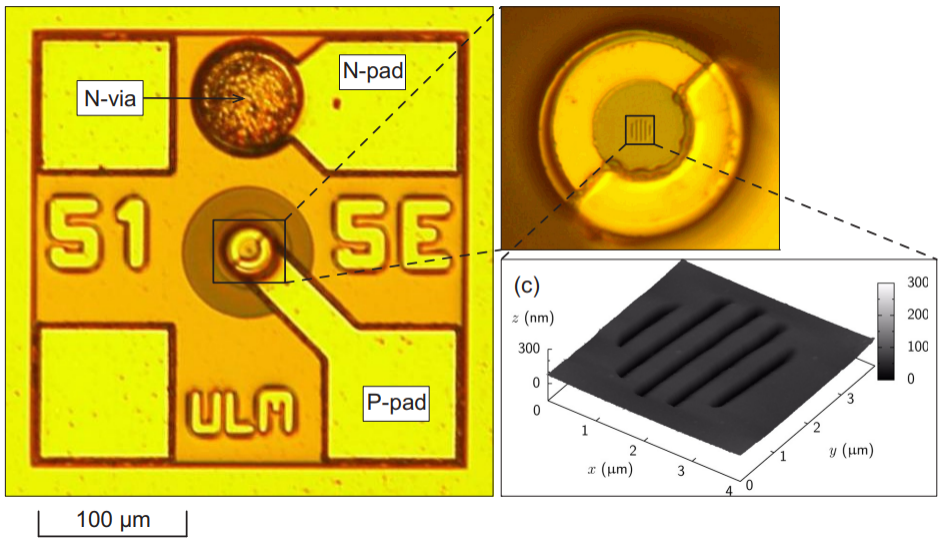
VCSEL fabricated by the University of Neuchâtel. Figure from [68].

The University of Ulm successfully fabricated single-mode and single-polarization VCSEL devices capable of operating at 85 °C by etching elliptical relief structures on the VCSEL surface, as shown in Figure 13 [60]. They then employed surface-relief gratings to develop 850 nm VCSELs, achieving a side-mode suppression ratio (SMSR) greater than 30 dB and an orthogonal polarization suppression ratio (OPSR) greater than 20 dB at 90 °C, with an output power exceeding 1 mW [61]. A subsequent technical report described the process of transferring an 850 nm VCSEL structure to devices emitting at 895 nm, a wavelength necessary for cesium-based atomic clocks [62,63]. Sandia National Laboratories and Mytek, LLC collaboratively developed high-performance VCSELs that achieved an SMSR greater than 35 dB with an injection current of 1 mA, and output power exceeding 1 mW at high temperatures [64]. The Rzhanov Institute of Semiconductor Physics, Siberian Branch, Russian Academy of Sciences (RAS) proposed 795 nm VCSEL devices suitable for Rb atomic clocks, with an SMSR close to 30 dB and an OPSR close to 40 dB at 80 °C [65]. The Ioffe Institute, RAS developed and experimentally tested VCSELs for a spectral range of 895 nm [66]. These VCSELs maintained stable single-mode output at high temperatures up to 90 °C and featured a fixed polarization direction. They provided a maximum output power exceeding 1 mW at temperatures ranging from 50 to 65 °C and a spectral linewidth less than 50 MHz. Unlike designs of polarization-stable VCSELs that utilize subwavelength gratings or photonic crystal structures in the output mirror, these newly developed VCSELs did not require lithography with submicron spatial resolution. The innovative laser source allowed for fine-tuning of the ^133^Cs D1 line wavelength and forming collimated output beams. The North China University of Technology and Tsinghua University collaboratively designed 780 nm VCSELs specifically for Coherent Population Trapping (CPT) atomic clocks. These VCSELs achieved a maximum output power close to 0.5 mW at 75 °C [67]. The University of Neuchâtel in Switzerland developed an 894.6 nm VCSEL for micro Cs atomic clocks based on CPT principles [68]. The VCSEL is shown in Figure 14 Princeton Optronics, USA, developed three single-frequency VCSELs specifically for atomic clocks, featuring lasing wavelengths of 780, 795, and 850 nm. These devices achieved single-frequency output powers of up to 40 mW [69]. CST in the UK developed an 894.5 nm VCSEL specifically tailored for CPT-based atomic clocks, achieving an SMSR exceeding 30 dB and an OPSR exceeding 15 dB. It also maintains superior performance at high temperatures, up to 70 °C [70]. The Beijing University of Technology developed 895 nm VCSELs specifically for Cs atomic clocks, achieving an output power of 0.86 mW, with SMSR greater than 20 dB [71].

**Figure 15:**
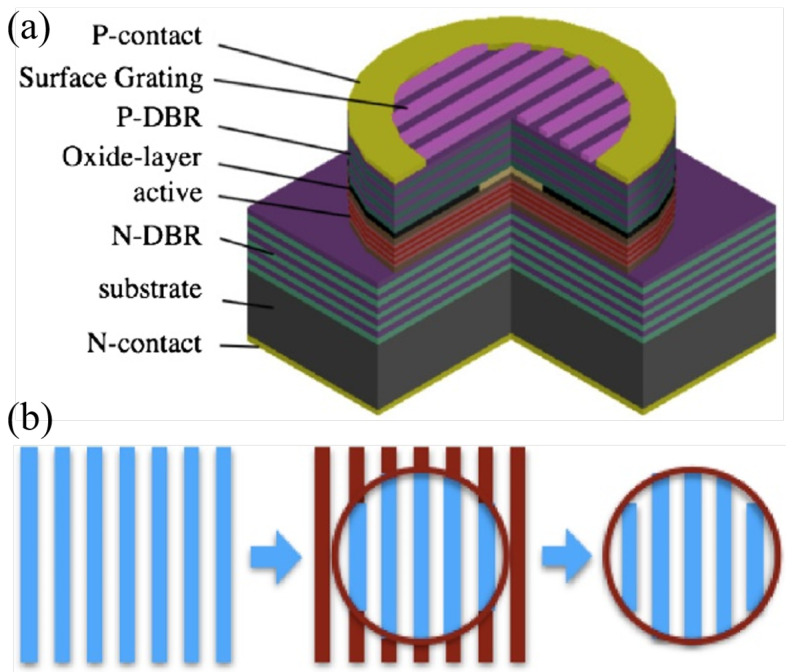
Shallow-surface grating VCSELs fabricated by CIOMP, CAS. (**a**) Schematic view of the surface grating VCSEL. (**b**) Patterns left in each step of the exposures. Figure from [76].

Efforts have been made to develop VCSELs for precise quantum measurements by Changchun Institute of Optics, Fine Mechanics and Physics, Chinese Academy of Sciences (CIOMP, CAS). By adjusting the position of gain spectrum and the cavity mode of oscillation cavity, the increase in power consumption of VCSEL at high temperatures has been effectively suppressed [72,73,74]. Through the integrated surface mode filter, the stable selection of the internal mode of VCSEL is realized [75], and an 894 nm VCSEL with a single-mode output power of more than 2 mW @ 92 °C is reported [52]. Also, they report the single-mode 795 nm VCSEL with a output power of more than 4 mW @ 80 °C, which has been applied in a nuclear magnetic resonance gyroscope system as the pump source [56]. The control of optical mode and polarization within VCSEL at the same time is also reported by them. As shown in Figure 15, a polarization-stable single-mode 894 nm VCSEL with an orthogonal polarization suppression ratio of 30 dB @ 80 °C is achieved using the shallow-surface gratings [76].

Due to the requirements for portability and low power consumption in many scenarios, there is a strong demand for VCSELs in the field of atomic magnetometers. Geometrics in the USA developed an all-optical scalar atomic magnetometer that utilizes VCSELs as pump sources. This magnetometer features no dead zones, high sensitivity, a high sampling rate, and high performance [77]. Shanghai Jiao Tong University employed VCSELs as pump light sources to significantly reduce the power consumption and size of atomic magnetometers. They developed a laser frequency stabilization method for a portable dual-cell cesium optical pump magnetometer utilizing VCSELs. This method included a laser temperature control circuit, an optical pump magnetometer signal feedback circuit, and a laser current source modulation and control circuit. The frequency stabilization, based on injection current modulation, was integrated into the dual-cell optical pump magnetometer design, achieving excellent stability and reducing stabilization costs. The temperature control accuracy achieved was 0.002 °C, and the wavelength stability was 4.36 × 10^−10^ over 10 s. This work preliminarily explored the potential for integrating and miniaturizing self-excited dual-cell optical pump magnetometers [78]. Nowadays, several technology companies, including QuSpin Inc. [79] and Beijing X-MAGTECH Technologies [80], have developed commercial atomic magnetometers that use VCSELs as the laser source.

### 3.3. Potentials of VCSELs

As an emerging technology, VCSELs still have a limited choice of parameters such as wavelength, mode, and power, especially for hot atomic device applications. However, there have been many efforts to optimize some of these parameters. These efforts are likewise highly instructive for the development of VCSELs for hot atomic devices.

Optical Power

One primary challenge of using VCSELs as pumping light sources for hot atomic devices is their limited optical power. Large aperture VCSELs and 2D VCSEL arrays may hold the key to the problem.

In 2001, the University of Ulm in Germany developed a 320 μm aperture VCSEL that produced a single-device output power of 0.89 W at a wavelength of 980 nm, while their two-dimensional array device achieved 1.55 W [81]. In 2005, Changchun Institute of Optical Machinery reported producing 500 μm aperture VCSEL single-tube devices capable of continuous output at room temperature, reaching 1.95 W [82]. In the same year, Princeton Optronics managed to achieve a continuous output of 3 W from a device with a 350 μm diameter by using a diamond heat sink [83].

The photon transport direction of VCSELs is the same as the epitaxial growth direction, making it easy to realize two-dimensional array integration. In 2008, Princeton Optronics achieved a power output of 45 W on a 5 mm × 5 mm VCSEL array [84]. Subsequently, Princeton Optronics designed an even more compact VCSEL array with a continuous power output of 231 W and a pulsed output of several kilowatts at a heat sink temperature of 15 °C, achieving the highest international level [85]. In 2019, Philips Photonics GmbH in Aachen, Germany, developed an optically pumped vertical external cavity surface emitting laser (OP-VECSEL). They demonstrated optical output power exceeding 10 W with an approximate diffraction-limited beam quality of M2~1.3 [86]. In China, the Changchun Institute of Optical Machinery (CIOM) of the Chinese Academy of Sciences (CAS) realized a 980 nm VCSEL single-tube pulse output of 92 W in 2011 [87], and the pulse output of a 4 × 4 VCSEL array reached 123 W. In 2014, the CIOM adopted four high-power VCSEL single-tube arrays in series, and the pulse output power was increased to 210 W [88].

**Figure 16:**
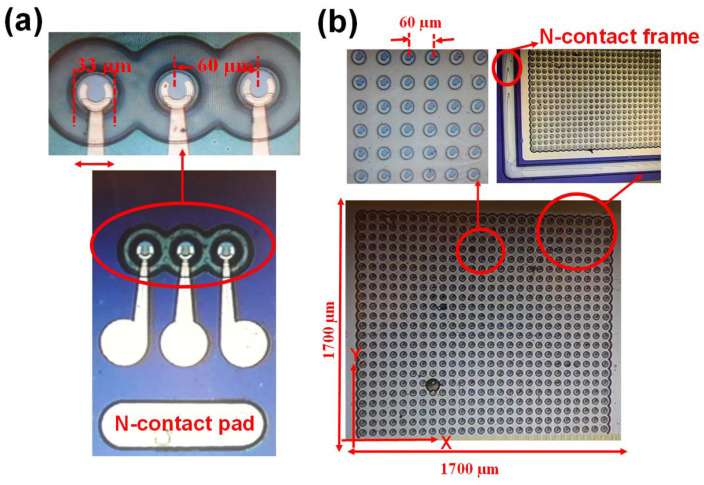
Top view of the (**a**) single reference VCSEL unit. (**b**) VCSEL array. Figure from [89].

The National Central University demonstrated a compact 7×7 coupled cavity VCSEL 850 nm array using a novel design for both the layout of the ultracompact array and the electrode pads, as shown in Figure 16. The demonstrated array shows heavier dampening of the electrical–optical (E–O) frequency response, a wider maximum 3 dB E–O bandwidth (17 vs. 13 GHz), and a Gaussian-like optical far-field pattern with a higher brightness output (65.95 vs. 40.8 kW cm^−2^ sr^−1^), under the same high output power (∼145 mW). The advantages of this novel VCSEL array lead to a much better quality of 32 Gbps with a higher brightness output [89,90].

Beam Quality

**Figure 17:**
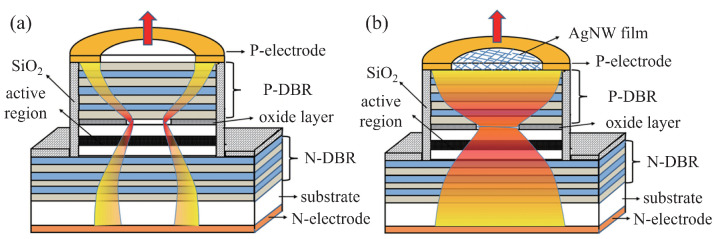
Schematic cross-sections of VCSELs with the illustration of the current flow in the devices: (**a**) VCSELs without AgNW film on the top surface, (**b**) VCSELs with AgNW film on the top surface. The current flow in the VCSELs with AgNW film on the top surface is more uniform. Figure from [91].

For large-aperture VCSELs, the uneven distribution of laser power density, caused by carrier accumulation and spatial hole burning effects, can significantly impact the output quality of the device [57]. One solution involves integrating optical components such as microlenses, diffractive optical elements (DOEs), and filters into the external cavity to control the high-power VCSEL optical field. However, the use of external optical elements compromises the device’s flexibility and is counterproductive to miniaturization efforts. Moreover, optimizing device structures can enhance power output and improve optical field distribution. These methods include applying silver nanowire thin films on the surface [91], creating surface-etched gratings [92,93,94], developing photonic crystal structures [95,96,97,98], and combining proton injection with oxide apertures [89]. As depicted in Figure 17, although silver nanowire thin films improve carrier uniformity, controlling the uniformity of nanowires across large areas remains challenging, which complicates the manufacturing and integration process and may introduce thermal coupling issues. Surface-etched gratings and photonic crystals enhance optical field control by increasing the loss of higher-order modes. However, their fabrication requires complex processes and precise control; otherwise, optical losses may increase, and optical output power may decrease, raising costs and limiting their applicability. While optimizing the size and distribution of proton injections can refine the optical field and yield higher beam quality, controlling the number and distribution of injected protons accurately is challenging. Otherwise, device performance degradation and reduced lifespan may occur [57].

**Figure 18:**
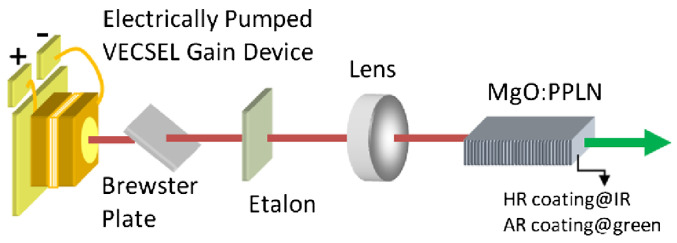
Experimental setup for the electrically pumped vertical external cavity surface emitting green laser. Figure from [100].

The resonant cavity of a VCSEL, composed of top and bottom DBRs, typically matches the wavelength order, leading to a broad divergence angle (half-angle width of approximately 15°) [99]. In 2014, to achieve a narrower divergence angle, vertical external cavity surface emitting lasers (VECSELs) emerged, as shown in Figure 18. The adoption of an external cavity significantly extends the cavity length from the wavelength scale to millimeters or even centimeters, effectively improving beam quality with a theoretical M2 close to 1 [100]. In 2022, the Institute of Physics of the Chinese Academy of Sciences demonstrated a topological cavity surface emitting semiconductor laser (TCSEL). This design utilizes a “Dirac vortex” topological optical cavity, currently the most advanced large-area single-mode optical cavity configuration available, theoretically capable of overcoming the traditional limitations faced by semiconductor lasers, where high output power and high beam quality could not be achieved simultaneously. A peak power of 10 W, a far-field divergence angle of less than 1°, and a 60 dB SMSR were achieved [101].

Linewidth

VCSELs typically exhibit a linewidth on the order of MHz, and there is still room for development in narrowing the linewidth and optimizing the structure [102]. Chongqing University has conducted research on VCSEL linewidth narrowing and polarization control, utilizing feedback from a whispering gallery mode microcavity resonator. This approach can narrow the VCSEL linewidth from 2.1 MHz to 32.6 kHz [103].

At the end of this section, we summarize VCSELs used in chip-scale hot atomic devices in Table 2.

### 3.4. Laser Heating

The laser heating method heats the vapor cell by irradiating it with laser light detuned from the alkali metal spectrum. Compared to the electric heating method, laser heating introduces no additional magnetic fields into the spin system, facilitating an all-optical configuration of atomic devices. The high collimation and energy concentration of laser light make it suitable for heating small-volume alkali vapor cells [106].

In 2009, Kitching’s group at NIST designed MEMS atomic magnetometers that utilized a 915 nm laser, detuned from the Rb atomic spectrum, for heating. The laser was delivered via fiber optics and directly irradiated the side wall of a rubidium vapor cell, which had a volume of just 12 mm^3^. With 200 mW of power, they successfully heated the rubidium vapor cell to 97 °C [107]. In 2012, the group further advanced this technique by using color filters to absorb specific wavelengths of light, employing a 1550 nm laser as the heat source to raise the atomic vapor cell temperature to 150 °C. In this work, 0.25 mm and 1 mm thick color filters were attached to the front and rear light transmission surfaces of the vapor cell, ensuring uniform absorption of the 1550 nm laser light and thus achieving uniform heating. To minimize thermal radiation and maximize temperature stability, the vapor cell was encapsulated in a silicon-glass frame within a vacuum environment, with low-outgassing epoxy resin used to secure the vapor cell to a 50 μm thick polyimide material [108]. In 2016, Los Alamos National Laboratory demonstrated a dual-beam fiber-coupled design with laser heating applied to atomic magnetometers, which achieved a sensitivity of 5 fT/Hz^1/2^ at low frequencies (50 Hz), higher than other fiber-coupled magnetometers, and can be improved to the sub-femtotesla level [109]. In 2023, ShanghaiTech University designed a magnetically heated structure based on laser heating principles. The theoretical heat transfer process was first modeled using Fourier heat transfer equations, followed by a study of the temperature distribution within the heating structure using the finite element method. The researchers analyzed the energy conversion and transfer mechanisms of the laser. To address the issue of poor thermal conductivity between the vapor cell and the heating chip, which led to uneven temperature distribution and low heating efficiency, graphite films were added to the four surfaces of the vapor cell. This modification reduced the thermal coefficient from 0.1308 to 0.0426, improving the heating performance [110].

Laser heating, excitation, and detection often utilize optical fibers to transmit laser beams to vapor cells, which can introduce challenges such as parasitic optical feedback. A portion of the laser beam is reflected into the fiber at an angle, and the reflected beam may interfere with the original beam, resulting in degradation of the beam quality and, in more serious cases, damage to the laser source. To mitigate this issue, anti-reflection coatings can be applied to both the inner and outer walls of the vapor cell, minimizing laser beam reflections and enhancing overall performance [111].

### 3.5. Optical Elements

**Figure 19:**
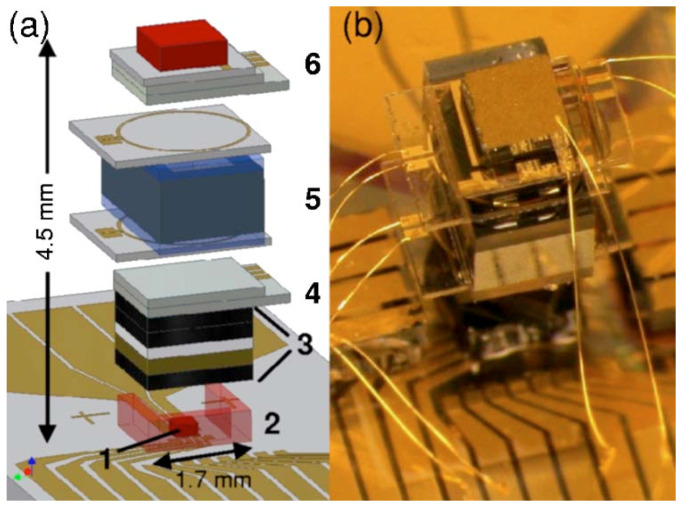
NIST’s chip-scale atomic magnetometer utilizing a 795 nm VCSEL. (**a**) Schematic of the magnetic sensor. The components are (1) VCSEL; (2) polyimide spacer; (3) optics package; (4) ITO heater; (5) ^87^Rb vapor cell with RF coils above and below it; and (6) ITO heater and photodiode assembly. (**b**) Photograph of the magnetic sensor. Figure from [112].

**Figure 20:**
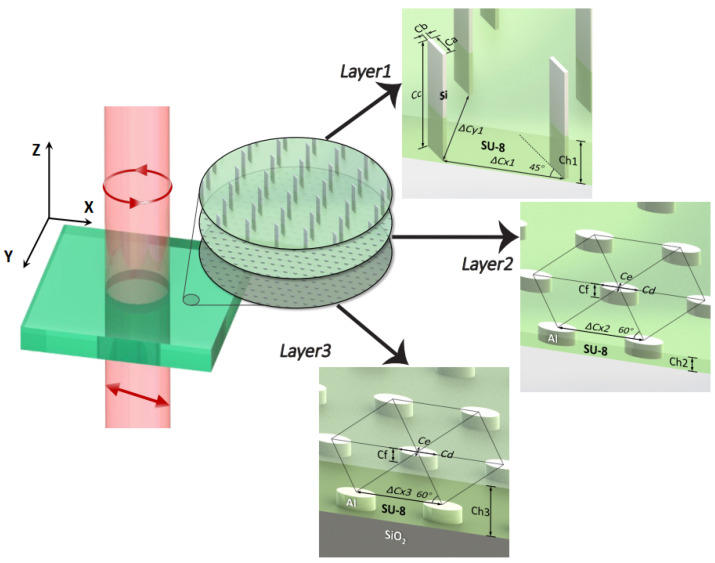
The fine structure of the circular polarization generator in the metasurface-based optical system for miniaturization of hot atomic magnetometers designed by ZJUT. Figure from [59].

In addition to the laser source, there are also various optical lenses and photodiodes in the optical system of a hot atomic device. These devices serve to regulate the power and polarization of the laser, and convert the optical signal into the electrical signal. Miniaturization of the laser optical path is also important to reduce the size of hot atomic devices. Figure 19 shows a chip-scale hot atomic magnetometer demonstrated by NIST [59]. The magnetic sensor is constructed by stacking the various components on a fused silica baseplate with patterned gold electrodes to bring electrical signals to and from the device. Laser light at 795 nm is derived from a VCSEL. Before entering the alkali vapor cell, the laser light passes through several optical elements that create circular polarization and attenuate the light to ∼10 μW. A linear polarizer is included to reject an unwanted laser mode orthogonal to the dominant mode and to ensure that the linear polarization of the light is properly aligned with the quarter wave plate. Next in the stack are the alkali vapor cell and ITO heaters. At the top of the stack, a p-i-n silicon photodiode detects the light transmitted through the vapor cell. The device occupies a volume of ∼25 mm^3^. In 2024, Zhejiang University of Technology (ZJUT) designed a metasurface-based optical system for the miniaturization of hot atomic magnetometers [112]. The optical systems contained highly efficient half-wave plates, polarizers, circular polarization generators, polarization-preserving reflectors, and polarizing beam splitters. Figure 20 shows the fine structure of the circular polarization generator. These components, compatible with semiconductor manufacturing, offer a promising solution for creating ultra-thin, compact atomic devices.

## 4. Coils

The coil systems are often indispensable for atomic devices, serving as electrical heaters or magnetic field generators.

Generally, in atomic precision measurement tasks, more atoms interacting with light leads to stronger measurement response capabilities. For chip-scale atomic devices, however, the cell volume is significantly limited. Heating the atomic vapor cell becomes the most common method for obtaining high atomic density. The vapor pressure *p*, which is the pressure of vapor in thermodynamic equilibrium with its liquid or solid form, depends on the temperature *T* at the liquid/solid–vapor interface. This relationship is succinctly described by the Clausius–Clapeyron equation:

(3) $$p\left(T\right)=p_{\infty }\exp\left(-\frac{L}{k_{B}T}\right),$$

where $p_{\infty }$ is a constant determined by the chemical properties of the substance, *L* represents the latent heat of fusion (for solids) or vaporization (for liquids), $k_{B}$ is the Boltzmann constant, and *T* is the absolute temperature, measured in Kelvin. The equation can be rewritten as:

(4) $$\log_{10}p=A-\frac{B}{T},$$

where *A* and *B* are material- (and phase-) dependent constants. The constants A and B corresponding to the different alkali metals are shown in Table 3. The number density *n* of the atomic vapor is given by:

(5) $$n\left(T\right)=\frac{p(T)}{k_{B}T}.$$

**Figure 21:**
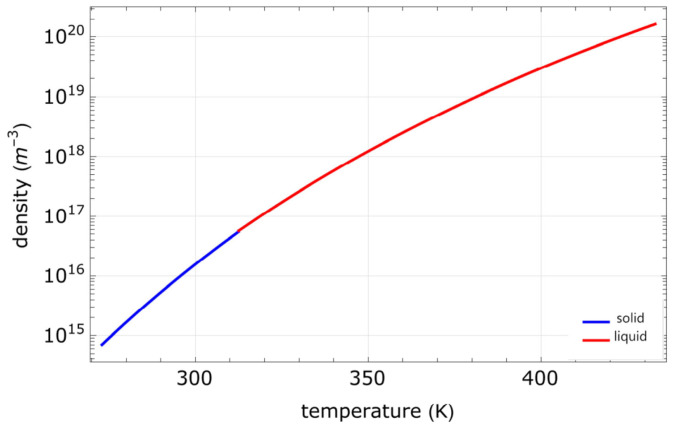
Relationship between the number density of rubidium atoms and temperature. Figure from [114].

The relationship between the number density of rubidium atoms and temperature is depicted in Figure 21. Near 370 K, the number density of rubidium atoms increases fivefold for every 10 K rise in temperature. Therefore, monitoring and stabilizing the vapor temperature or atomic density is crucial for maintaining signal stability. The atomic number information can typically be obtained by using thermistors, by measuring the resistance of the heater windings, or by assessing the optical density of the atomic vapors. It is worth noting that the probe chamber is often heated to a higher temperature than the reservoir chamber. This temperature gradient helps prevent the deposition of alkali metals on the light-passing surfaces.

Electrical heating is the predominant method used for heating hot atomic devices. Its advantages include high heating power and precise temperature control, making it highly suitable for chip-scale hot atomic devices and experimental platforms. In this method, the vapor cell is heated by the thermal effect of coil resistance. To mitigate the electromagnetic effects of heating currents on the atomic system, the frequency of the heating current is typically set much higher than the operating frequency of the atomic system. Additionally, to minimize the influence of the magnetic fields generated by electrical heating, heating elements are often designed to counteract magnetic interference. This is accomplished by either twisting the heating wires in pairs or configuring them into a double-layer twisted pattern on a membrane. Such configurations ensure that the magnetic fields generated by the currents in the heating wires oppose each other, effectively cancelling out. Given that changes in cell temperature can alter atomic source parameters and the strength of detection signals, it is crucial for the heating device not only to avoid magnetic field interference but also to offer precise temperature control [115]. The electrical heating method facilitates temperature stabilization through feedback control of the heating current, providing a convenient and effective solution for maintaining consistent operating conditions.

In addition to the purpose of heating, coils are also employed to generate the DC and AC magnetic fields necessary for controlling atomic spin dynamics. Spatial homogeneity is an important criterion for generated magnetic fields. For example, DC magnetic field gradients can cause decoherence effects in atomic spins, and AC magnetic field inhomogeneity could result in incoherent excitation of atomic spins in the atomic ensemble. The uniform requirement of the magnetic field adds a great deal of complexity to designing and manufacturing chip-level micro magnetic coils, suffering from drawbacks such as manufacturing difficulties and poor repeatability [12].

Traditionally, coil structures in atomic devices were wire-wound, but they have gradually been replaced by printed circuit board (PCB) coils. In 2008, the University of California, in collaboration with the National Institute of Standards and Technology, used printed circuit coil winding technology to fabricate micro magnetic field coils in NMRG [116]. More recently, in 2021, Southeast University employed stacked PCBs to construct magnetic compensation coils for SERF magnetometers [117]. The latest advancements in coil structures leverage MEMS technology. MEMS is a miniature system that integrates microscopic electronic components, mechanical components, and miniature sensors. MEMS coils are particularly noted for their compact size and compatibility with other electrical components, making them widely used in chip-scale hot atomic devices. The main fabrication processes used in MEMS include thin-film deposition, photolithography, and etching, with common substrate materials being silicon, silicon nitride, and glass. Using a MEMS coil with glass as the substrate and indium tin oxide (ITO) as the wiring material as an example, the fabrication process primarily involves the following steps [118]:

1. Glass substrate cleaning: Take the quartz glass with a length and width of 70 mm and a thickness of 0.55 mm, clean the surface of the glass substrate with an ultrasonic cleaner and deionized water, and dry it with a drying oven.
2. ITO magnetron sputtering coating: Evenly coat the cleaned glass substrate with a layer of ITO (film thickness of about 200 nm).
3. Coating photoresist: The use of homogenizer in the glass coated with ITO side should be evenly coated with a layer of photoresist, photoresist model AZ1500.
4. Photolithography + development + etching: The production of a good pattern mask, the use of model URE-2000/35 photolithography in the photoresist engraved with the desired pattern, with the ZX-238 positive gel developer to remove the exposed area, leaving the unexposed area, and then soaked in 20% of dilute hydrochloric acid etching is not covered by the photoresist area.
5. De-gumming: The use of AZ400T de-gumming solution to remove excess photoresist.
6. Point coating conductive silver glue: The use of high concentrations of quick-drying conductive silver glue connected to the upper and lower pins.
7. Buckle insulation: The two pieces of surface circuitry engraved on the surface of the mutual buckle and the use of transparent insulating high-temperature double-sided tape to prevent short circuits.
8. Lead electrode: from the glass surface pins with conductive silver paste lead to the positive and negative poles.

### 4.1. Rigid MEMS Coils

Commonly used coils are non-transparent and cannot be attached directly to the vapor cell flux surface, hindering the integration of chip-scale hot atomic devices. In contrast, transparent coils made from glass and ITO can be directly affixed to the light-emitting surface of the vapor cell. This feature is particularly advantageous for heating and generating magnetic fields in chip-scale vapor cells. In 2007, Schwindt et al. at NIST designed an ITO heater utilizing laser etching to remove parts of the ITO from a glass substrate to create a pattern, resulting in a 120 mm wide ITO strip. A layer of benzocyclobutene insulation was deposited in the middle. The ITO heater was constructed with upper and lower layers bonded together using conductive epoxy resin, with the patterns on both layers mirrored. This configuration cancels out the magnetic field generated by the current in the lower ITO layer with the return magnetic field in the upper ITO layer directly above it. The winding path of the current through the heater yields a high resistance of up to 2 kΩ, effectively reducing the current and dissipating power across most of the heater area [59]. Then the same group further optimized this approach by employing a 20 kHz AC current to heat a rubidium atomic cell to approximately 190 °C, achieving an alkali metal atomic number density of $6\times 10^{14}\;{\mathrm{cm}}^{-3}$ [119]. In 2010, NIST conducted research on SERF atomic magnetometers using a heating-measurement process with a period of 4 s [45]. This heating method is favored for its simplicity and ease of implementation. However, it does not support continuous magnetic field measurement, which limits both the bandwidth and the temperature control accuracy. In 2015, Southeast University demonstrated the manufacturing and characteristics of wafer-level micro silicon heaters for chip-scale atomic magnetometers. A series of processes, including dry etching and anodic bonding of highly doped silicon and Pyrex glass wafers, were used to form micro silicon heaters and optical channels. By applying an AC current of 180 kHz, the cell was heated to around 150 °C [120]. In 2019, North China University of Science and Technology developed a digital temperature control system for MEMS atomic cells, using a microcontroller and a proportional–integral–derivative (PID) algorithm. They fabricated a specialized ITO heating glass for MEMS atomic cells through magnetron sputtering, achieving a temperature control accuracy of 0.10 °C [121]. In 2021, the same institution engineered single-layer and double-layer structured micro weak magnetic electric heating chips, based on principles of electric heating and inverse magnetic compensation. Initially, the principle of magnetic field cancellation was analyzed using Biot–Savart law theory. Subsequently, double-layer chips were produced using MEMS technology. Testing indicated that the magnetic flux density generated by the current at a distance of 5 mm from the chip was 0.0722 nT/mA [122]. In 2023, Peter the Great St. Petersburg Polytechnic University unveiled a thin-film heating device fabricated using MEMS technology. This device incorporates two thin-film components—a heater and a temperature sensor—both situated on a glass substrate in a planar circular and curved configuration. The manufacturing process begins with the deposition of a 100 nm thick chromium film onto the surface of a Corning 0211 glass chip using magnetron sputtering. This is followed by photolithography and etching to create the first conductive layer. The next step involves depositing a silicon dioxide insulation layer using magnetron sputtering, repeating the initial process to establish the second conductive layer, and linking them with gold vias. Experiments demonstrated that the relationship between magnetic flux density and the heating current in the heating wire is linear, exhibiting a coefficient of 2.1 nT/mA [123].

Harbin Engineering University implemented a differential wiring method for heating coils, utilizing microfabrication film technology to create square, pure copper non-magnetic heating coils on a ceramic substrate. They employed COMSOL Multiphysics simulation software to analyze the distribution of additional steady-state magnetic fields produced by the coils under a 2.2 mA DC condition. The university developed a non-magnetic heater capable of 3 W heating power and 0.1 °C temperature control accuracy. Experimental results demonstrated that the instantaneous magnetic disturbance generated by the heater is 2.24 pT [124].

### 4.2. Flexible MEMS Coils

In 2012, the University of Wisconsin–Madison incorporated a Minco double-layer twisted heating film design in its magnetometer. Two heating films with the same wiring shape are connected back-to-back in series, effectively reducing the magnetic field generated during heating. The resultant magnetic field during operation is limited to the picotesla range, sufficient to heat a rubidium atomic cell to temperatures between 140 and 180 °C [125]. In 2015, Beihang University proposed a four-layer helical heating film, first theoretically analyzing the single-layer and multi-layer conductive modes, then analyzing the role of multi-layer films in canceling out the magnetic field at the bottom, and finally simulating experiments to minimize the magnetic field. For the four-layer heating film, the magnetic flux density introduced in the z-direction by the current is 8.4 pT/mA [126]. In 2017, Beihang University arranged the heating wire in a parallel adjacent form and fixed it to a high-temperature resistant material substrate to form a heating film. High-temperature resistant materials were used as the substrate, and non-magnetic nickel–chromium alloy wire was used as the heating wire, with a resistance of approximately 150 Ω for the heating film. Through PID control, a short-term stability of ±5 mK and a long-term stability of ±10 mK were achieved, and the magnetic field noise introduced by the non-magnetic electrical heating system of the alkali metal cell was less than 15 fT/Hz^1/2^ [127]. In 2018, the Agency for Defense Development of South Korea explored a specially designed double-layer polyimide film heater for use in atomic spin gyroscope (ASG) experiments. This heater utilizes a non-magnetic copper–nickel alloy wire, with a double-layer structure configured to have opposite current directions to mitigate magnetic field generation. The publication details the technical specifications of the heater design and its applications in ASG experiments, emphasizing the importance of precise temperature control and minimal magnetic field interference [128].

### 4.3. Other Types of Electric Heaters

In 2013, the University of California, Irvine, developed micro magnetic coils using glass-blowing technology, achieving a magnetic field gradient of 5%/cm. However, this three-dimensional coil could only be used for spherical cells [129]. Then they introduced a manufacturing process for a MEMS component consisting of a folded Helmholtz coil and an integrated cell heater. The fabrication process starts with a 500 μm silicon wafer coated with 3000 Å of LPCVD silicon nitride. First, a layer of Cr/Au is deposited, and Cr TFE and Au GE8110 etchants are used for wet metal etching to complete the preparation of the first layer. Subsequently, a layer of 14 μm polytetrafluoroethylene is deposited over the metal and then etched using reactive ion etching (RIE) to form flexible connections. The second layer is formed using the same method to create the trajectory of the Helmholtz coil. Finally, the coil and connections are prepared on the back according to the RIE-DRIE-RIE sequence of the Si_3_N_4_-Si-Si_3_N_4_ layer. The article further explores the relationship between the magnetic field generated along the coil’s axis and the coil’s dimensions and uniformity [130].

In 2018, Southeast University introduced a wafer-level integration method for micro heaters and vapor cells in chip-scale SERF magnetometers. This method utilized magnetron sputtering technology to prepare micro heaters on the surface of vapor cells. They employed a mixture of polysilicon and aluminum as the material and optimized the resistivity by varying the ratio of polysilicon to aluminum in the sputtering target material. Conductive adhesive was used to attach the leads to the micro heaters. The performance of the micro heaters was tested, and experiments were conducted to evaluate their performance under different voltages. By heating rubidium vapor to 120 °C with high-frequency AC current, a sensitivity of 6.3 pT/Hz^1/2^ was achieved for the magnetometer [131]. In 2023, Southeast University proposed a novel MEMS atomic chip with single-layer heating functionality. Additionally, by laterally shifting the heaters on the chip from the detection area, the influence of heating noise on magnetometer performance was effectively suppressed. Experimental results demonstrated that compared to atomic chips heated with stationary heaters, moving the heaters reduced sensor noise by over 90%, while increasing sensor sensitivity by more than twelvefold [132].

In 2022, China Shipbuilding Group Co., Ltd. designed a non-magnetic heater and non-magnetic constant temperature control circuit using MEMS technology with a symmetrical four-wire structure. The non-magnetic constant temperature control circuit further reduced constant magnetic field interference through AC heating. Using a field-programmable gate array as the core, high-frequency heating signals were generated using direct digital frequency synthesis technology, followed by power amplification for non-magnetic constant temperature control. Test results showed that the temperature noise peak-to-peak value of the cell constant temperature control could reach 0.02 °C [133].

In 2023, the China Academy of Engineering Physics leveraged MEMS technology to design a non-magnetic heating coil for a small-scale nuclear magnetic resonance gyroscope, featuring a millimeter-scale atomic cell and a “+–+-++-” double-layer wire layout. Thermal-magnetic simulations were conducted to analyze the impact of the heating coil’s structural parameters and heating current on both the temperature of the atomic cell and the residual magnetic field. The study also examined how wire spacing, insulation layer thickness, and wire material influence the internal residual magnetic field and temperature within the atomic cell. With four-sided heating and a 6.3 mA DC heating current, the average temperature of the cell reached 170.95 °C, with a residual magnetic field of 0.256 nT under DC conditions, and a maximum residual magnetic field inside the atomic cell of less than 0.3 nT [134].

### 4.4. Coils for Uniform Magnetic Fields

The uniformity of the magnetic field generated by magnetic coils is crucial for the performance of hot atomic devices. It is essential to not only refine the miniaturization process for coil fabrication but also to optimize the methods for achieving magnetic field uniformity. The Barcelona Institute of Science and Technology in Spain has demonstrated miniature coils for three-dimensional localized field control for atomic magnetometers. The coils are designed on biplanar surfaces using a stream-function approach and then fabricated using standard printed-circuit techniques. They applied this technology to a laboratory-scale optically pumped magnetometer, achieving a sensitivity of approximately 20 fT/Hz^1/2^. They also demonstrated a coil set measuring 7 × 17 × 17 mm^3^, specifically optimized for magnetoencephalography, where multiple sensors operate in close proximity. Characterization of the field profile using ^87^Rb free-induction spectroscopy and other techniques showed greater than 96% field homogeneity over the target volume of a MEMS vapor cell and a compact stray-field contour of approximately 1% at 20 mm from the center of the cell [135].

## 5. Conclusions and Discussion

In conclusion, the advancement of chip-scaled hot atomic devices represents a significant stride in the realm of quantum precision measurement. These devices integrate the delicate quantum properties of atomic systems into compact formats, making quantum measurement techniques more accessible and applicable across a broader range of disciplines and industries.

Throughout this review, we have explored the key technological breakthroughs and engineering innovations that have driven the miniaturization and enhanced functionality of hot atomic devices. From the development of microfabricated components like MEMS-based coils and heaters to the integration of sophisticated optical systems such as VCSELs, each advancement has contributed to more stable, accurate, and efficient devices.

The future of chip-scaled hot atomic devices in quantum precision measurement appears very promising. However, to transform these devices into practical tools for quantum precision measurement, the technologies discussed in this review must be further advanced. Progress in these areas is often interlinked, heavily relying on continual enhancements and innovations in micromanufacturing techniques. The development of new, specialized materials is also crucial in this field. For instance, nanocrystalline magnetic shielding techniques are gaining increasing attention. Using nanocrystalline materials as magnetic shields in chip-scale atomic devices shows significant promise [136].

## Figures and Tables

**Table 1:** Parameters of some 795 nm commercial edge-emitting laser diodes.

Manufacturer	Series	Package	Wavelength (nm)	Linewidth (MHz)	Power (mW)	SMSR (dB)	OPSR (dB)
Thorlabs	DBR795PN	Butterfly	795	1	40	50	16
Photodigm	PH795DBR	Butterfly/TO	795	0.7	20–180	30	19
Toptica Eagleyard	EYP-DFB-0795	Butterfly	795	0.6	40–80	30	-
Sacher Lasertechnic	DFB-0795-080	TO	795	1.5	80	-	-

**Table 2:** Performance parameters of VCSELs used in chip-scale hot atomic devices.

Institute	Year	Wavelength (nm)	Linewidth (MHz)	Power (mW)	SMSR (dB)	OPSR (dB)	Temperature (°C)
NIST [58]	2000	852	50	0.87	-	-	31.2
NIST [59]	2007	795	-	0.01	-	-	-
Uni-Ulm [61]	2005	850	-	3.2	40	24	-
Uni-Ulm [62]	2008	895	-	8	25	-	-
Uni-Ulm [63]	2013	895	-	0.9	40	21	23
SNL [64]	2007	795, 850	-	3	-	15	85
ISP SB RAS [65]	2009	795	-	0.2	30	-	-
Ioffe Institute, RAS [66]	2018	895	50	1	-	20	65
NUC [67]	2009	795	-	0.5	-	-	75
UNINE [68]	2013	895	20	0.7	42	22.7	23
Princeton Optronics [69]	2015	780, 795, 850	-	40	50	20	-
CST [70]	2019	895	50	1	33	15	70
BJUT [71]	2020	895	-	0.86	20	-	-
CAS [72]	2013	795	-	1	-	-	72
CAS [73]	2014	795	0.3	17	-	-	52
CAS [74]	2015	895	-	-	25	-	110
CAS [75]	2017	895	-	0.45	30	-	80
CAS [76]	2018	894	-	0.55	-	32	80
CAS [52]	2022	894	-	2.02	29.2	20	92
CAS [56]	2022	795	-	4.1	41.68	27.4	80
II-VI Laser [104]	2012	795	-	1	20	10	50
Vixar [105]	2014	895	-	0.1	20	16	80

**Table 3:** Constants *A* and *B* of different alkali metals [113].

	Melting Point (°C)	Solid		Liquid	
		*A*	*B*	*A*	*B*
Cs	28.5	4.711	3999	4.165	3830
Rb	39.3	4.857	4215	4.312	4040
K	63.5	4.961	4646	4.402	4453
Na	97.8	5.298	5603	4.704	5377

## Abbreviations

The following abbreviations are used in this manuscript:

CSAC	Chip-scale atomic clock
DAVLL	Dichroic atomic vapor laser lock
DBR	Distributed Bragg reflector
DFB	Distributed feedback
FPC	Flexible printed circuits
ITO	Indium tin oxide
MEMS	Micro-electro-mechanical system
NMRG	Nuclear magnetic resonance gyroscope
OPM	Optical pump magnetometer
OPSR	Orthogonal polarization suppression ratio
PID	Proportional–integral–derivative
SERF	Spin-exchange relaxation-free
VCSEL	Vertical-cavity surface-emitting laser
